# Characterizing the Racial Discrepancy in Hypoxemia Detection in VV-ECMO: An ELSO Registry Analysis

**DOI:** 10.21203/rs.3.rs-3617237/v1

**Published:** 2023-11-17

**Authors:** Andrew Kalra, Christopher Wilcox, Sari D Holmes, Joseph E Tonna, In Seok Jeong, Peter Rycus, Marc M Anders, Akram M Zaaqoq, Roberto Lorusso, Daniel Brodie, Steven P. Keller, Bo Soo Kim, Glenn J R Whitman, Sung-Min Cho

**Affiliations:** Johns Hopkins University School of Medicine; Mercy Hospital of Buffalo; Johns Hopkins University School of Medicine; University of Utah Health; Chonnam National University Hospital; Extracorporeal Life Support Organization; Baylor College of Medicine; University of Virginia; Maastricht University Medical Centre (MUMC); Johns Hopkins University School of Medicine; Johns Hopkins University School of Medicine; Johns Hopkins University School of Medicine; Johns Hopkins University School of Medicine; Johns Hopkins University School of Medicine

**Keywords:** pulse oximetry, arterial blood gas, venovenous extracorporeal membrane oxygenation, racial/ethnical disparities, hypoxemia

## Abstract

**Importance:**

Skin pigmentation influences peripheral oxygen saturation (SpO_2_) measured by pulse oximetry compared to the arterial saturation of oxygen (SaO_2_) measured via arterial blood gas analysis. However, data on SpO_2_-SaO_2_ discrepancy are limited in venovenous-extracorporeal membrane oxygenation (VV-ECMO) patients.

**Objective:**

To determine whether there is racial/ethnical discrepancy between SpO_2_ and SaO_2_ in patients receiving VV-ECMO. We hypothesized VV-ECMO cannulation, in addition to race/ethnicity, accentuates the SpO_2_-SaO_2_ discrepancy due to significant hemolysis.

**Design:**

Retrospective cohort study of the Extracorporeal Life Support Organization Registry from 1/2018–5/2023.

**Setting:**

International, multicenter registry study including over 500 ECMO centers.

**Participants:**

Adults (≥ 18 years) supported with VV-ECMO with concurrently measured SpO_2_ and SaO_2_ measurements.

**Exposure:**

Race/ethnicity and ECMO cannulation.

**Main outcomes and measures:**

Occult hypoxemia (SaO_2_ ≤ 88% with SpO_2_ ≥ 92%) was our primary outcome. Multivariable logistic regressions were performed to examine whether race/ethnicity was associated with occult hypoxemia in pre-ECMO and on-ECMO SpO_2_-SaO_2_ calculations. Covariates included age, sex, temporary mechanical circulatory support, pre-vasopressors, and pre-inotropes for pre-ECMO analysis, plus single-lumen *versus* double-lumen cannulation, hemolysis, hyperbilirubinemia, ECMO pump flow rate, and on-ECMO 24h lactate for on-ECMO analysis.

**Results:**

Of 13,171 VV-ECMO patients (median age = 48.6 years, 66% male), there were 7,772 (59%) White, 2,114 (16%) Hispanic, 1,777 (14%) Black, and 1,508 (11%) Asian patients. The frequency of on-ECMO occult hypoxemia was 2.0% (*N* = 233). Occult hypoxemia was more common in Black and Hispanic *versus* White patients (3.1% *versus* 1.7%, *P* < 0.001 and 2.5% *versus* 1.7%, *P* = 0.025, respectively).

In multivariable logistic regression, Black patients were at higher risk of pre-ECMO occult hypoxemia *versus* White patients (adjusted odds ratio [aOR] = 1.55, 95% confidence interval [CI] = 1.18–2.02, *P* = 0.001). For on-ECMO occult hypoxemia, Black patients (aOR = 1.79, 95%CI = 1.16–2.75, *P* = 0.008) and Hispanic patients (aOR = 1.71, 95%CI = 1.15–2.55, *P* = 0.008) had higher risk *versus* White patients. Furthermore, higher pump flow rate (aOR = 1.29, 95%CI = 1.08–1.55, *P* = 0.005) and higher on-ECMO 24h lactate (aOR = 1.06, 95%CI = 1.03–1.10, *P* < 0.001) significantly increased the risk of on-ECMO occult hypoxemia.

**Conclusions and Relevance:**

Hispanic and Black VV-ECMO patients experienced occult hypoxemia more than White patients. SaO_2_ should be carefully monitored during ECMO support for Black and Hispanic patients especially for those with high pump flow and lactate values at risk for occult hypoxemia.

## Introduction

Arterial blood gas (ABG) analysis is the gold-standard method to measure arterial oxygen saturation (SaO_2_) as it directly measures the partial pressure of oxygen in arterial blood.^1^ Pulse oximetry is a standard non-invasive continuous method of monitoring peripheral oxygen saturation (SpO_2_) via measurement of hemoglobin saturation through spectroscopy. Darker skin pigmentation may worsen occult hypoxemia (previously defined as SaO_2_ ≤ 88% despite SpO_2_ ≥ 92%^2^) as was seen in a cohort of 10,001 intensive care unit (ICU) patients where Black patients experienced occult hypoxemia 3-fold more than White patients.^3^ In a study of respiratory failure patients 6 hours before extracorporeal membrane oxygenation (ECMO) cannulation, the frequency of pre-ECMO occult hypoxemia in Black patients was higher than in White patients.^2^ However, this study analyzed only pre-ECMO oxygen saturation values and did not account for clinically-relevant covariates such as hemolysis or presence of vasopressor usage.

In addition to skin pigmentation bias, SpO_2_ is also unable to distinguish between oxygen bound to hemoglobin (oxyhemoglobin), carbon monoxide bound to hemoglobin (carboxyhemoglobin) or oxidized hemoglobin (methemoglobin or sulfhemoglobinemia), which can further cause inaccurate pulse oximetry readings.^4–6^ This is particularly important in ECMO patients, where accurate oxygenation measurements are critical and their complex physiology^7^ can further influence the accuracy of these measurements. Specifically, high ECMO pump flow rate and larger cannula size^8^ can cause hemolysis due to mechanical shearing of red blood cells,^9–11^ leading to formation of carboxyhemoglobin and thus causing inaccurate SpO_2_ measurements. This phenomenon was observed in a cohort of 40 venovenous (VV)-ECMO patients; however, this study is limited by small sample size and did not account for ECMO-relevant covariates or race/ethnicity in their analyses.^12^

We sought to address these previous limitations using concurrently measured SpO_2_ and SaO_2_ data points within a multicenter, international cohort of ECMO patients, to examine for discrepancy by race/ethnicity. In addition, we hypothesized that ECMO-induced hemolysis would worsen occult hypoxemia in VV-ECMO patients. Finally, as the single-lumen cannula may require greater resistance of perfusion and overall pump flow to maintain adequate perfusion, we also hypothesized that single *versus* double-lumen cannulation strategies may impact the SpO_2_-SaO_2_ discrepancy.

## Methods

### Study design and population

This study was approved by the Johns Hopkins Hospital Institutional Review Board with a waiver of informed consent, as this was a retrospective observational study (IRB00216321). The Extracorporeal Life Support Organization (ELSO) registry is an international multicenter registry from over 500 ECMO centers globally^13^. The registry collects demographic information, pre-ECMO comorbidities, pre-ECMO and on-ECMO laboratory and hemodynamic information, on-ECMO complications, and outcomes.^14^ Comorbidity information was recorded using *International Classification of Diseases, 10th Revision (ICD-10)* codes.

We included patients who were 1) 18 years of age or older; 2) supported with VV-ECMO; and 3) had data on race/ethnicity. We excluded repeat ECMO runs within individual patients to reduce bias, patients without data on hypoxemia at either time points, and patients with extreme outlier values for the difference between SpO_2_ and SaO_2_. Multiple imputation was not used as most variables did not meet appropriate criteria.

### Data collection

The ELSO registry collects ABG and hemodynamic information prior to and after ECMO cannulation (i.e., “pre-ECMO” and “on-ECMO”, respectively). Pre-ECMO ABGs were drawn no later than 6 hours prior to ECMO cannulation, and pre-ECMO ventilator settings were recorded within 6 hours of ECMO cannulation. If multiple ABGs existed within a specific duration, the pre-ECMO ABG that was closest to the start of ECMO cannulation was chosen. On-ECMO ABGs were drawn after ECMO cannulation began, no later than 30 hours after cannulation. If multiple ABGs existed, the on-ECMO ABG closest to 24 hours after the start of cannulation was selected. On-ECMO hemodynamics were collected closest to 24 hours after ECMO cannulation, though they could be collected at 18–30 hours. SpO_2_ and SaO_2_ data were abstracted by a trained ELSO data manager/abstracter from each center and were pulled simultaneously.

### Definitions

SpO_2_ was defined as the noninvasive pulse oximeter measured oxyhemoglobin saturation while SaO_2_ was the percent arterial blood oxyhemoglobin saturation from ABG. Pre-ECMO ventilator type settings included conventional ventilation, high-frequency oscillatory ventilation, other high frequency ventilation (high frequency jet ventilation or percussive ventilation), other non-specified ventilations, and no ventilation. Pre-ECMO mechanical circulatory support included intra-aortic balloon pump, Impella^®^, and left ventricular assist device. Pre-ECMO vasopressor infusions included dopamine, epinephrine, norepinephrine, phenylephrine, and vasopressin. Pre-ECMO inotrope infusions included dobutamine, enoximone, levosimendan, milrinone, nicardipine, nitroglycerin, nitroprusside, and tolazoline. Infusions were treated as a binary covariate (i.e., the presence or absence of the infusion). Pre-ECMO vasopressor and inotrope infusions were employed for at least 6 hours within 24 hours of the start of ECMO cannulation. By definition, single-lumen cannulation involves placement in two vascular access sites and double-lumen cannulation involves placement of a cannula in single vascular access site.

On-ECMO complications included ECMO mechanical circuit failure, renal replacement therapy, hemolysis, hyperbilirubinemia, cardiac arrhythmia, and gastrointestinal hemorrhage. Definitions for each are provided in the **eMethods**.

Race/ethnicity was coded per patient as one of: Asian, Black, Hispanic, Middle Eastern or North African, Native American, Native Pacific Islander, Multiple, Other, Unknown, and White. We restricted our primary analysis to Asian, Black, Hispanic, and White patients. White patients were chosen as the reference comparator based on previous literature showing the first pulse oximeters were calibrated to this race/ethnicity.^15,16^

### Outcomes

Occult hypoxemia was defined as SaO_2_ ≤ 88% with a time-matched SpO_2_ ≥ 92%.^2^ The primary outcomes were the occult hypoxemia (binary variable) and SpO_2_-SaO_2_ discrepancy (continuous variable), which were compared between different races/ethnicities. We also assessed the accuracy and precision for SpO_2_ to predict SaO_2_.

### Statistical Analysis

Continuous variables were assessed for normality with the Kolmogorov-Smirnov test, and all were determined to be not normally distributed. Therefore, these variables are denoted as median with interquartile range (IQR). Categorical variables are represented as frequency with percentages. The Kruskal-Wallis, Wilcoxon rank-sum, and Pearson chi-square tests were utilized to compare continuous and categorical variables, respectively. SpO_2_-SaO_2_ differences were compared with Kruskal-Wallis and Wilcoxon rank-sum tests. A *P* value < 0.05 was considered statistically significant (**eMethods** contain more details).

Bland-Altman analyses were used to assess agreement between SpO_2_ and the gold standard, SaO_2_, by calculating the difference between SpO_2_ and SaO_2_, the mean of SpO_2_ and SaO_2_, the estimated bias (median difference) and limits of agreement (median and 95% limits of agreement at 2.5th and 97.5th percentiles) using a nonparametric method to estimate the limits given the non-normality of the differences between SpO_2_ and SaO_2_.^17,18^ The one-sample Wilcoxon signed-rank test was used to compare the median difference between SpO_2_ and SaO_2_ (estimated bias) for Asian, Black, and Hispanic patient groups as compared to the median value for White patients (hypothetical value set at 0). Boxplots were performed to visually assess the SpO_2_-SaO_2_ discrepancy while scatterplots were used to visually assess the correlation between SpO_2_ and SaO_2_. Spearman correlations were conducted for pre-ECMO and on-ECMO SpO_2_ and SaO_2_ by race/ethnicity.

Multivariable logistic regressions were performed to examine whether race/ethnicity was associated with occult hypoxemia in pre-ECMO and on-ECMO measurements. One pre-ECMO SpO_2_-SaO_2_ pair and a corresponding on-ECMO SpO_2_-SaO_2_ pair was measured from the same patient for adequate comparison. We selected covariates a priori that were hypothesized to be associated with the SpO_2_-SaO_2_ discrepancy based on clinical judgement and prior data. Covariates in both the pre-ECMO and on-ECMO models included age, sex, presence of pre-ECMO temporary mechanical circulatory support (tMCS), and presence of pre-ECMO vasopressor and inotrope infusions. The on-ECMO model also included hemolysis, hyperbilirubinemia, cannulation strategy (single-*versus* double-lumen), ECMO pump flow rate, and on-ECMO serum lactate value. Adjusted odds ratios (OR) are presented with 95% confidence intervals (CIs).

As a sensitivity analysis, multivariable linear regression was used to assess the on-ECMO SpO_2_-SaO_2_ discrepancy as a continuous variable by race/ethnicity. The model included covariates selected a priori based on clinical judgement and prior data: age, sex, cannulation strategy, hemolysis, on-ECMO lactate, pre-ECMO tMCS, ECMO duration, pre-ECMO inotrope infusions, and pre-ECMO ventilation strategy.

Receiver-operating characteristic curve (ROC) analyses were performed to determine the accuracy of on-ECMO SpO_2_ in predicting SaO_2_. Based on previous literature,^19–22^ the following on-ECMO SaO_2_ thresholds were tested: 88%, 92%, and 95%. All SpO_2_ values were tested to detect the specific SaO_2_ threshold at or below its value. Area under the receiver-operating characteristic curve (AUC), sensitivity, and specificity were calculated. In addition, the AUC for each SaO_2_ threshold was compared by race/ethnicity groups. All statistical analyses were performed using R Studio (R 4.1.2, www.r-project.org) or IBM SPSS Statistics for Windows, Version 28.0 (IBM Corp, Armonk, NY).

## Results

### Study population

**Figure 1:**
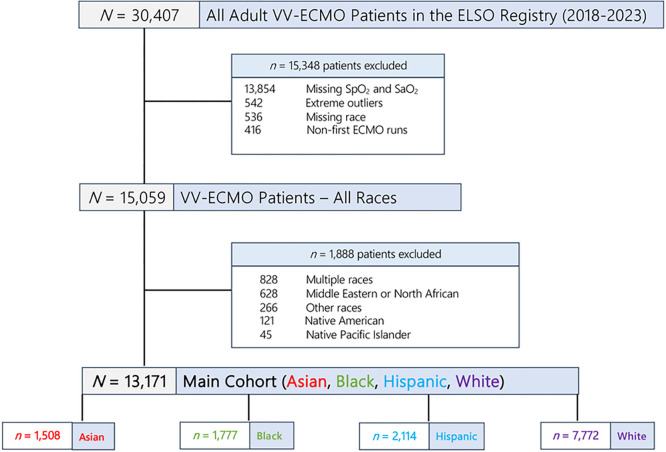
Creation of study cohort from the ELSO Registry.

Of 30,407 VV-ECMO patients, we included 13,171 patients for our study after applying the inclusion and exclusion criteria (Fig. 1). Of 13,171 VV-ECMO patients (median age = 48.6 years, 66% male), 7,772 (59%) were White, 2,114 (16%) were Hispanic, 1,777 (14%) were Black, and 1,508 (11%) were Asian (Table 1). Of 9,851 patients with valid COVID-19 data, 59% (*N* = 5,830) had a diagnosis of SARS CoV-2 and COVID-19.

The median ECMO duration was 11.4 days (IQR = 5.7–22.9). Of patients with complete cannulation information (*N* = 12,966), 9,833 (76%) received single-lumen VV-ECMO *versus* 3,133 (24%) received double-lumen VV-ECMO. Asian patients were more likely to have single-lumen-cannulation strategy (*N* = 1,279, 88%) whereas Black patients were less likely to have single-lumen cannulation (*N* = 1,206, 69%) compared to other races/ethnicities.

### Occult Hypoxemia

Overall, on-ECMO occult hypoxemia was observed in 2.0% of patients (233 of 11,709). On-ECMO occult hypoxemia was more common in Black (3.1% vs 1.7%, *P* < 0.001) and Hispanic (2.5% vs 1.7%, *P* = 0.025) patients *versus* White patients. The proportion of on-ECMO occult hypoxemia was similar between Asian and White patients (1.6%% vs 1.7%, *P* = 0.787).

**Figure 2:**
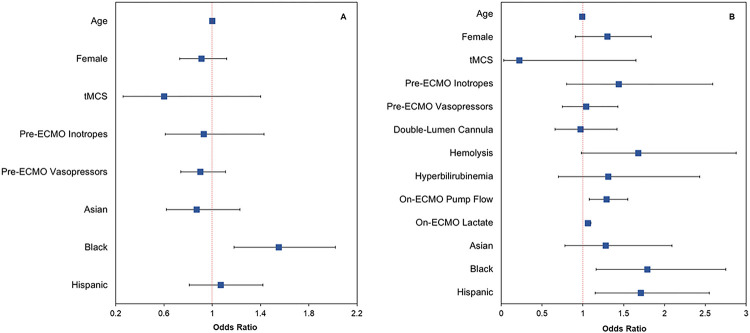
Forest plots of odds ratios and 95% confidence intervals from the multivariable logistic regression for occult hypoxemia in **A)** pre-ECMO SpO_2_-SaO_2_ and **B)** on-ECMO SpO_2_-SaO_2_ pairs. Pre-ECMO and on-ECMO SpO_2_-SaO_2_ measurements were extracted from the same patient.

In a multivariable logistic regression, Black patients were at higher risk of pre-ECMO occult hypoxemia *versus* White patients (aOR = 1.55, 95%CI = 1.18–2.02, *P* = 0.001; Fig. 2A, **eTable 1**). No other race/ethnicity group differences were found.

For on-ECMO occult hypoxemia, multivariable logistic regression analysis found that Black patients (aOR = 1.79, *P* = 0.008) and Hispanic patients (aOR = 1.71, *P* = 0.008) had greater risk *versus* White patients (Table 2, Fig. 2B). Other significant risk factors included pump flow rate at 24 hours on ECMO (aOR = 1.29, *P* = 0.005) and on-ECMO lactate (aOR = 1.06, *P* < 0.001; Table 2).

### SpO_2_-SaO_2_ Discrepancy

**Figure 3:**
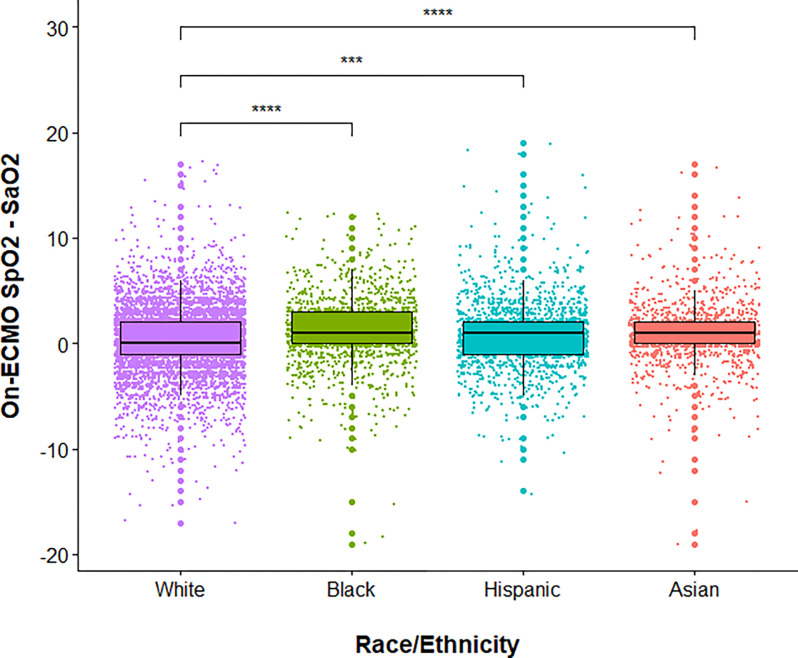
Boxplots showing pulse oximetry (SpO_2_) overestimates arterial blood gas (SaO_2_) in Black, Asian, and Hispanic venovenous (VV)-extracorporeal membrane oxygenation (ECMO) patients, compared to White VV-ECMO patients. Purple color = White patients. Green color = Black patients. Red color = Asian patients. Turquoise color = Hispanic patients. Solid black line represents the median value while the upper and lower limits of the boxes represent the 75% and 25% quartiles, respectively. White patients were used as the reference comparator. **** and *** represent *P* values <0.0001 and <0.001, respectively.

Figure 3 depicts boxplots of the on-ECMO SpO_2_-SaO_2_ difference for each race/ethnicity. The overall on-ECMO estimated bias for the entire cohort was 1.0 (median difference between SpO_2_ and SaO_2_) with 95% limits of agreement at −6.0 and 7.0 (2.5th and 97.5th percentiles). In Bland-Altman plots, the limits of agreement for Asian, Black, and Hispanic patients were wider as compared to White patients (**eFigure 1**). In addition, the median difference of the SpO_2_-SaO_2_ difference for Asian, Black, and Hispanic patients was significantly higher than the hypothetical value set at the median for White patients. The overall Spearman correlation coefficient comparing on-ECMO SpO_2_ with SaO_2_ was moderate (*r*_*s*_=0.69, *P* < 0.001). The Spearman correlation coefficients comparing on-ECMO SpO_2_ with SaO_2_ were relatively comparable in magnitude across the race/ethnicity groups, and all were significant (*P* < 0.001): Asian patients (*r*_*s*_=0.69), Black patients (*r*_*s*_=0.67), Hispanic patients (*r*_*s*_=0.72), and White patients (*r*_*s*_=0.68), but Fisher r-to-z transformation did find a significant difference in *r*_*s*_ between White and Hispanic patients (z=−2.84, *P* = 0.005).

### Sensitivity Analysis for SpO_2_-SaO_2_discrepancy

A multivariable linear regression was performed to corroborate the results of the primary logistic regression analysis for on-ECMO occult hypoxemia, using the continuous variable of the difference between on-ECMO SpO_2_ and SaO_2_. After adjusting for the covariate set (*F* = 3.3, *P* < 0.001), the addition of race/ethnicity to the model was associated with a significant *r*^2^ change (0.01, *P* < 0.001). Each race/ethnicity group was found to have greater on-ECMO discrepancies as a continuous variable as compared with White patients after adjustment for the covariates (**eTable 2**). The double-lumen cannula was also found to have greater on-ECMO discrepancies as a continuous variable as compared to single-lumen cannula.

### On-ECMO Receiver-operating characteristic curve analyses

At an 88% SaO_2_ threshold, SpO_2_ predicted hypoxemia with an AUC of 0.89 (95%CI = 0.88–0.90), at a 92% threshold, SpO_2_ predicted hypoxemia with an AUC of 0.87 (95%CI = 0.86–0.88), and at a 95% threshold, SpO_2_ predicted hypoxemia with an AUC of 0.83 (95%CI = 0.83–0.84; **eFigure 2**), sensitivity of 0%, and specificity of 100%. There were no significant race/ethnicity group differences in AUC for SpO_2_ at an 88% SaO_2_ threshold between White patients (AUC = 0.90) and Asian (AUC = 0.88), Black (AUC = 0.89), and Hispanic (AUC = 0.88) patients. At a 92% SaO_2_ threshold, the AUC was smaller for Black patients (AUC = 0.85) *versus* White patients (AUC = 0.88, *P* = 0.037), but not for Asian (AUC = 0.86) or Hispanic (AUC = 0.86) patients *versus* White patients. No significant race/ethnicity group differences in AUC for SpO_2_ at an 95% SaO_2_ threshold were found between White patients (AUC = 0.83) and Asian (AUC = 0.84), Black (AUC = 0.83), and Hispanic (AUC = 0.85) patients.

### Exploratory Analysis: Single-lumen versus Double-lumen

There was no difference in the incidence of on-ECMO occult hypoxemia for single-lumen *versus* dual-lumen cannulation (2% vs 2%, *P* = 0.527). However, the correlation between on-ECMO SpO_2_ and SaO_2_ was weaker for single-lumen (*r*_*s*_=0.68, *P* < 0.001) *versus* double-lumen (*r*_*s*_=0.72, *P* < 0.001) using Fisher r-to-z transformation to compare *r*_*s*_ values (*z*=−3.45, *P* < 0.001).In patients with confirmed hemolysis, there was no difference in the incidence of on-ECMO occult hypoxemia for single-lumen *versus* double-lumen cannulation (6% vs 4%, *P* = 0.463). The correlation between on-ECMO SpO_2_ and SaO_2_ was weaker for single-lumen (*r*_*s*_=0.67, *P* < 0.001) versus double-lumen (*r*_*s*_=0.83, *P* < 0.001) using Fisher r-to-z transformation to compare *r*_*s*_ values (*z* = −4.28, *P* < 0.001).

### Exploratory Analysis: COVID-19 vs non-COVID-19

In those with COVID-19 (N = 5,830, 59%), the incidence of pre-ECMO occult hypoxemia did not differ for patients with and without COVID-19 (4% vs 4% *P* = 0.993), but the incidence of on-ECMO occult hypoxemia was significantly greater for patients with COVID-19 (2.4% vs 1.5%, *P* = 0.003).

## Discussion

In this retrospective observational analysis of the international multicenter ELSO Registry, we found that the frequency of unadjusted and adjusted occult hypoxemia in VV-ECMO patients was higher in Black and Hispanic patients *versus* White patients. After adjusting for clinically relevant risk factors, we found that ECMO support exacerbated occult hypoxemia (compared to pre-ECMO). This finding suggests that, in addition to race/ethnicity, being supported by VV-ECMO may lead to more inaccurate pulse oximetry measurements likely mediated by greater ECMO pump flow and corresponding associated hemolysis.

### Pre-ECMO vs On-ECMO Occult Hypoxemia

While a previous ELSO Registry study using solely pre-ECMO SpO_2_ and SaO_2_ measurements found a similar frequency of occult hypoxemia in Hispanic patients compared to White patients,^2^ we found a higher frequency of occult hypoxemia in Hispanic *versus* White patients in “*on*-ECMO” SpO_2_ and SaO_2_ values. This finding suggests the SpO_2_-SaO_2_ discrepancy is worse during the time that patients were supported with ECMO. Besides one single-center study analyzing this discrepancy in 57 VV-ECMO patients,^22^ which found the unadjusted frequency of occult hypoxemia to be greater in Black *versus* White patients but not in Hispanic or Asian *versus* White patients, no other literature exists pertaining to this discrepancy directly for patients supported on ECMO.

Undetected hypoxemia during cannulation is important as ECMO patients are already at high-risk for many complications such as acute brain injury from inadequate oxygen delivery, which increases mortality.^23,24^ Occult hypoxemia may increase in-hospital mortality due to increased risk of organ dysfunction,^25,26^ lung injury,^27^ and worse neurocognition.^28^ Accordingly, monitoring for occult hypoxemia in patients with such risk factors (Black and Hispanic race/ethnicity, high pump-flow and lactate) in ECMO is extremely important.

Additionally, compared to this pre-ECMO ELSO study, our study has several strengths including 1) having a larger sample size (N = 13,171 *versus* N = 1,562), 2) the use of methodically rigorous statistical methods such as the Kolmogorov-Smirnov test to assess for non-normality and nonparametric analyses when comparing the SpO_2_-SaO_2_ discrepancy, and 3) the adjustment of more clinically-relevant and ECMO-specific covariates versus only adjusting for measured SpO_2_ and sex as was done in this prior study.

### Cannulation strategy, ECMO pump flow rate, and hemolysis

In contrast to previous data,^7^ we found no differences in the SpO_2_-SaO_2_ discrepancy between patients with single-lumen *versus* double-lumen, perhaps because the rates of hemolysis in our population were similar (5.5% *versus* 5.4%) which may be the primary driver of increased SpO_2_-SaO_2_ discrepancy via carbon monoxide production increasing carboxyhemoglobin. However, we still observed the correlation between SpO_2_ *versus* SaO_2_ was weaker in single-lumen *versus* double-lumen, with and without confirmed hemolysis. However, relative to single-lumen, the double-lumen cannula enhanced the SpO_2_-SaO_2_ discrepancy in our multivariable linear regression analysis. Overall, further research is warranted to investigate the association between cannulation strategy and the SpO_2_-SaO_2_ discrepancy.

Although “ELSO-defined” hemolysis during ECMO was not independently associated with occult hypoxemia, higher ECMO pump flow rates were associated with higher occurrence of occult hypoxemia. This finding may indicate varying degree of hemolysis is occurring with high ECMO pump flow rates.^7,8,29^ Due to shear mechanical stress that is produced by high pump flow rates in combination with large cannula sizes^8^ throughout the ECMO circuit and oxygenator, ECMO-associated hemolysis can occur.^30^ Furthermore, intravascular hemolysis, which has been reported to occur in 18% of ECMO-supported patients, can increase plasma hemoglobin.^31^ This hemoglobin can then scavenge endothelial-derived nitric oxide, which causes oxidative stress and triggers inflammatory signaling pathways that can potentially worsen the discrepancy as well.^29,32^

### Limitations

In line with previous studies,^2,3,33,34^ race/ethnicity was used as a substitute for skin tone as clinicians do not obtain this information, which likely does not completely reflect the heterogeneity of skin pigmentation found within races/ethnicities. The ELSO registry is an international consortium of over 500 ECMO centers; therefore, heterogeneity in data collection and the patient cohort inherently exists. However, this diversity in sampling may be offset by the large sample size of our study. Frequency of occult hypoxemia was relatively low in our cohort; however, it was measured at a single timepoint during ECMO cannulation and may not reflect the SpO_2_-SaO_2_ discrepancy throughout the entire ECMO run, as observed in previous studies.^7,22^ Additionally, prospective observational studies are needed to assess causation effects.

## Conclusions

Black and Hispanic VV-ECMO patients are at higher risk for occult hypoxemia and overestimated true oxygen saturation measurements compared to White patients. Patients who were supported with ECMO had an increased risk of the SpO_2_-SaO_2_ discrepancy compared to the pre-ECMO SpO_2_-SaO_2_ discrepancy, which was greater in non-White patients. As occult hypoxemia and SpO_2_-SaO_2_ discrepancy may worsen in patients with ECMO support, clinicians should carefully monitor ABGs during ECMO support for those with such risk factors.

## Figures and Tables

**Table 1:** Baseline characteristics and clinical variables of VV-ECMO patients stratified by race/ethnicity.

	Total (*N* = 13,171)	Asian (*n* = 1,508, 11%)	Black (*n* = 1,777, 14%)	Hispanic (*n* = 2,114, 16%)	White (*n* = 7,772, 59%)
**Demographics**					
Age (years)	48.6 (37.0–58.3)	49.8 (38.7–60.7)	44.3 (32.4–55.0)	44.9 (35.4–54.5)	50.2 (38.3–59.5)
Male	8,675 (66)	1,029 (68)	1,021 (58)	1,519 (72)	5,106 (66)
Body Mass Index, kg/m^2^	30.7 (26.0–36.4)	25.6 (22.9–29.7)	32.5 (26.5–38.7)	31.1 (27.1–36.2)	31.1 (26.5–36.9)
**Year ECLS**					
2018	1,626 (12)	217 (14)	197 (11)	120 (6)	1,092 (14)
2019	2,096 (16)	296 (20)	250 (14)	198 (9)	1,352 (17)
2020	3,756 (29)	472 (31)	537 (30)	881 (42)	1,866 (24)
2021	3,909 (30)	377 (25)	514 (29)	683 (32)	2,335 (30)
2022	1,576 (12)	137 (9)	250 (14)	206 (10)	983 (13)
2023	208 (2)	9 (1)	29 (2)	26 (1)	144 (2)
**Past medical history**					
Diabetes	1,372 (10)	96 (6)	242 (14)	316 (15)	718 (9)
Hypertension	1,873 (14)	112 (7)	331 (19)	285 (13)	1,145 (15)
Atrial fibrillation	845 (6)	36 (2)	108 (6)	94 (4)	607 (8)
Cardiomyopathy	177 (1)	10 (1)	40 (2)	27 (1%)	100 (1)
COPD	478 (4)	20 (1)	46 (3)	21 (1)	391 (5)
COVID-19 status (*N* = 9,851)	5,830 (59)	621 (59)	722 (53)	1,433 (79)	3,054 (54)
**Pre-ECMO support**					
tMCS	310 (2)	23 (2)	71 (4)	17 (1)	199 (3)
Vasopressor Infusions	8,119 (62)	992 (66)	1,059 (60)	1,194 (57)	4,874 (63)
Inotrope Infusions	870 (7)	82 (5)	144 (8)	113 (5)	531 (7)
**Pre-ECMO mean blood pressure (mm Hg)**	77 (69–88)	78 (69–90)	78 (69–90)	80 (71–89)	76 (68–87)
**Pre-ECMO ABG**					
pH	7.28 (7.19–7.36)	7.28 (7.19–7.36)	7.27 (7.17–7.35)	7.29 (7.20–7.37)	7.28 (7.18–7.36)
HCO_3_- (mEq/L)	26.6 (22.1–31.4)	25.0 (21.0–29.6)	26 (22.1–30.2)	28.0 (24.0–34.0)	26.6 (22.0–31.2)
PaO_2_	67 (56–84)	65 (56–80)	68 (53–87)	68 (57–84)	68 (56–84)
PaCO_2_	59 (48–74)	58 (46–74)	59 (47–74)	60 (49–75)	59 (48–73)
Lactate (mmol/L)	1.8 (1.2–3.0)	1.9 (1.2–3.2)	1.8 (1.2–3.4)	1.6 (1.2–2.4)	1.7 (1.2–3.0)
SpO_2_	91 (85–95)	90 (84–94)	91 (84–96)	91 (86–95)	91 (86–95)
SaO_2_	91 (85–95)	90 (84–94)	90 (83–95)	91 (85–95)	91 (85–95)
**On-ECMO mean blood pressure (mm Hg)**	76 (70–85)	79 (71–88)	77 (71–86)	77 (70–85)	75 (69–83)
**On-ECMO pulse pressure (mm Hg)**	55 (46–66)	52 (43–63)	55 (45–68)	56 (47–67)	56 (46–67)
**On-ECMO ABG**					
pH	7.40 (7.36–7.44)	7.40 (7.36–7.46)	7.40 (7.36–7.44)	7.41 (7.37–7.45)	7.40 (7.36–7.44)
HCO_3_- (mEq/L)	27 (24–31)	26 (23–29)	26 (23–29)	28 (24–32)	27 (24–31)
PaO_2_	78 (66–100)	83 (68–107)	80 (67–109)	75 (63–94)	78 (66–98)
PaCO_2_	44 (38–50)	42 (36–48)	43 (38–48)	44 (39–51)	44 (39–50)
Lactate (mmol/L)	1.5 (1.1–2.2)	1.6 (1.2–2.4)	1.5 (1.1–2.2)	1.4 (1.1–2.0)	1.5 (1.0–2.2)
SpO_2_	96 (93–98)	97 (94–99)	97 (93–99)	95 (92–98)	96 (93–98)
SaO_2_	95 (92–98)	96 (93–98)	96 (93–98)	95 (91–97)	95 (93–97)
**Pump Flow (4 hours)**	4.10 (3.60–4.65)	3.95 (3.40–4.36)	4.15 (3.65–4.70)	4.13 (3.68–4.70)	4.15 (3.60–4.70)
**Pump Flow (24 hours)**	4.14 (3.60–4.72)	3.95 (3.40–4.42)	4.12 (3.60–4.72)	4.20 (3.70–4.79)	4.20 (3.65–4.80)
**Cannulation Strategy (N = 12,966)**					
Single-Lumen (Two Sites)	9,833 (76)	1,279 (88)	1,206 (69)	1,572 (75)	5,776 (75)
Double-Lumen (One Site)	3,133 (24)	169 (12)	545 (31)	525 (25)	1,894 (25)
**Days on ECMO support**	11.4 (5.7–22.9)	11.3 (5.8–23.2)	10.1 (5.3–21.2)	15.8 (7.6–30.2)	10.7 (5.3–21.0)
**ECMO complications**					
ECMO circuit mechanical failure	1,224 (9)	138 (9)	156 (9)	237 (11)	693 (9)
Renal replacement therapy	3,419 (26)	368 (24)	483 (27)	490 (23)	2,078 (27)
Hemolysis	659 (5)	44 (3)	80 (5)	155 (7)	380 (5)
Hyperbilirubinemia	534 (4)	56 (4)	69 (4)	114 (5)	295 (4)
Cardiac arrhythmia	1,202 (9)	93 (6)	156 (9)	165 (8)	788 (10)
Gastrointestinal hemorrhage	790 (6)	92 (6)	112 (6)	156 (7)	430 (6)

Abbreviations: ECMO, extracorporeal oxygenation membrane; tMCS, temporary mechanical circulatory support; VV, venovenous.

**Table 2:** Factors associated with on-ECMO occult hypoxemia frequencies in multivariable logistic regression analysis.

	OR	95% CI	*P* value
Age	0.99	0.98–1.00	0.069
Female sex	1.30	0.91–1.84	0.147
Race/ethnicity			
White	*Reference*		
Black	1.79	1.16–2.75	0.008
Asian	1.28	0.78–2.09	0.332
Hispanic	1.71	1.15–2.55	0.008
Pre-ECMO support			
Pre-ECMO tMCS	0.22	0.03–1.65	0.142
Pre-ECMO inotrope infusions	1.44	0.80–2.59	0.228
Pre-ECMO vasopressor infusions	1.04	0.75–1.43	0.830
ECMO physiology			
Double-lumen cannula (one-site)	0.97	0.66–1.42	0.874
Hemolysis	1.68	0.98–2.88	0.060
Hyperbilirubinemia	1.31	0.70–2.43	0.402
Pump flow at 24 hours on-ECMO	1.29	1.08–1.55	0.005
On-ECMO lactate	1.06	1.03–1.10	<0.001

Abbreviations: CI, confidence interval; ECMO, extracorporeal membrane oxygenation; OR, odds ratio; tMCS, temporary mechanical circulatory support.

Pre-ECMO tMCS consisted of intra-aortic balloon pump, Impella, and left ventricular assist devices. Pre-ECMO inotropic infusions consisted of dobutamine, enoximone, levosimendan, milrinone, nicardipine, nitroglycerin, nitroprusside, tolazoline. Pre-ECMO vasopressor infusions included dopamine, epinephrine, norepinephrine, phenylephrine, and vasopressin. Hemolysis was defined as a peak plasma hemoglobin of at least 50 mg/dL occurring at least once during the ECMO run and sustained for at least 2 consecutive days. Hyperbilirubinemia was defined as a total bilirubin >10 mg/dL, conjugated bilirubin >3 mg/dL, or need for extracorporeal purification for elevated bilirubin.

## Key Points

### Question:

Do race/ethnicity and VV-ECMO cannulation influence the discrepancy between oxygen saturation measured by SpO_2_ *versus* SaO_2_?

### Findings:

In a retrospective cohort study of 13,171 VV-ECMO patients from the Extracorporeal Life Support Organization Registry (1/2018–5/2023), Black and Hispanic patients experienced *on*-ECMO occult hypoxemia more frequently than White patients. The adjusted frequency of occult hypoxemia was greater in *on*-ECMO *versus pre*-ECMO SpO_2_-SaO_2_ measurements; high pump flow rates and lactate values increased the risk of *on*-ECMO occult hypoxemia.

### Meaning:

SaO_2_ should be carefully monitored during ECMO support for Black and Hispanic patients especially for those with high pump flow and serum lactate.

## Abbreviations

ABG: arterial blood gas
aOR: adjusted odds ratio
AUC: area under the receiver-operating characteristic curve
CI: confidence interval
COVID-19: Coronavirus Disease 19
ECMO: extracorporeal membrane oxygenation
ELSO: Extracorporeal Life Support Organization
IQR: interquartile range
PaO_2_: partial pressure of oxygen
ROC: receiver-operating characteristic
SaO_2_: oxygen saturation measured by arterial blood gas
SpO_2_: oxygen saturation measured by pulse oximetry
SD: standard deviation
tMCS: temporary mechanical circulatory support
VV-ECMO: venovenous ECMO
